# The Influence of Cannabis and Alcohol Use on Sexuality: An Observational Study in Young People (18–30 Years)

**DOI:** 10.3390/healthcare10010071

**Published:** 2021-12-31

**Authors:** Pablo Roman, Ana Ortiz-Rodriguez, Ana Romero-Lopez, Miguel Rodriguez-Arrastia, Carmen Ropero-Padilla, Nuria Sanchez-Labraca, Lola Rueda-Ruzafa

**Affiliations:** 1 Department of Nursing, Physiotherapy and Medicine, Faculty of Health Sciences, University of Almeria, 04120 Almeria, Spain; pablo.roman@ual.es (P.R.); lrr606@ual.es (L.R.-R.); 2 Health Research Centre CEINSA, University of Almeria, 04120 Almeria, Spain; 3 Research Group CTS-451 Health Sciences, University of Almeria, 04120 Almeria, Spain; 4 Almeria Health District, 04008 Almeria, Spain; anaortizrguez@gmail.com; 5 Hospital de Poniente, 04700 Almeria, Spain; anaromero.4@hotmail.com; 6 Pre-Department of Nursing, Faculty of Health Sciences, Jaume I University, 12071 Castellon de la Plana, Spain; arrastia@uji.es (M.R.-A.); ropero@uji.es (C.R.-P.); 7 Research Group CYS, Faculty of Health Sciences, Jaume I University, 12071 Castellon de la Plana, Spain

**Keywords:** alcohol consumption, cannabis, epidemiology, observational study, sexuality, young adult

## Abstract

The consumption of cannabis and alcohol results in a variety of effects on the psychic functions of young users. Notwithstanding their widespread and prevalent use, the impact of these drugs on sexual health remains unknown. Thus, the aim of this study is to analyse the influence of alcohol and cannabis consumption on sexual function in young people. An observational study was conducted in 274 participants aged 18–30 years. The following selection tools were used: Alcohol Use Disorders Identification Test, Cannabis Abuse Screening Test (CAST), and Changes in Sexual Functioning Questionnaire Short-Form. Participants who were at high risk of having cannabis-related problems performed better on the CAST concerning sexual function, arousal, and orgasm. Participants at high risk had higher arousal and orgasm scores than those who were not at risk for cannabis problems. Improvements in sexual function were found between people who were at high risk of having alcohol problems and those who were not at risk. Sexual function in young people who use cannabis and alcohol more frequently was shown to be better than in those who do not use either, highlighting the need for more information aimed at the young population.

## 1. Introduction

The use of addictive substances, primarily for recreational purposes, such as alcohol, tobacco, and cannabis, remains a major health issue among young people [1,2,3], with significant short- and long-term health implications including dependence, cardiovascular disease, respiratory changes, emphysema, and cancer [4,5,6]. A common reason for drug consumption is in the context of sexual relations. According to a survey, regardless of gender or sexual preference, alcohol is the most commonly used substance, followed by cannabis and 3,4-methylenedioxymethamphetamine (MDMA) to enhance the sexual experience. However, the impact of these drugs on sexual function is still uncertain. The evidence is inconsistent, with contradictory reports of both benefits and harms [7].

Alcohol, a depressant of the nervous system, is related to the facilitation of sexual behaviour and arousal [8,9], but different studies have found dose-dependent negative effects on potency and sexual capacity [10]. Alcohol interferes with sexual responses due to metabolic changes since it alters nitric oxide synthesis. Nitric oxide participates in the physiology of erection by inducing the relaxation of cavernous smooth muscle, resulting in vasodilation of the penile arteries and, as a result, an erection [11]. However, the relationship between alcohol consumption and the risk of sexual dysfunctions such as erectile dysfunction or changes in vaginal pulse amplitude is still being debated [10,12]. Cannabis, on the other hand, appears to have sex-dependent effects, as cannabis consumption before sexual interaction enhances desire, improves orgasm, and reduces discomfort in women [13], whereas frequent cannabis use in men renders it difficult to reach orgasm [14]. Indeed, chronic use of cannabis mildly modulates semen and hormonal parameters, such as a higher level of follicle-stimulating hormone, which is indicative of infertility [15].

Despite these controversial implications, both positive and detrimental, the amount of research that has investigated the influence of cannabis and alcohol use on sexual function is still limited, even more with validated surveys. Therefore, the aim of this study is to analyze the impact of alcohol and cannabis use on sexual function in young people aged 18 to 30 years old.

## 2. Materials and Methods

### 2.1. Design

An observational study was conducted in Almeria (Spain) from January to June 2020 in accordance with the reporting guidelines of the Strengthening the Reporting of Observational Studies in Epidemiology (STROBE) criteria [16].

### 2.2. Population and Participants

Convenience sampling was used to select our participants. In addition, snowball sampling was used to increase the sample size. The selection criteria included participants who were between the ages of 18 and 30, agreed to participate voluntarily, and were either regular cannabis or alcohol users or non-users. Individuals who used substances other than cannabis and alcohol were excluded, as were participants who had any diagnosis (for example, depression or diabetes) that could influence their sexuality, to ensure that the results of our study were not influenced.

### 2.3. Data Collection

#### 2.3.1. Variables and Data Collection Tools

Table 1 shows the variables and data collection tools that were used.

#### 2.3.2. Sociodemographic Variables

An ad hoc questionnaire was prepared with the following items: sex, age, sexual orientation (heterosexual, homosexual, bisexual, or other), and level of education (none, primary, secondary, high school, university or intermediate or higher).

#### 2.3.3. Outcome Variables

Sexual function and problems associated with cannabis and alcohol were measured as outcome variables.

**⚜** Problems associated with alcohol were assessed through the Spanish version of the Alcohol Use Disorders Identification Test (AUDIT) [20], which consists of 10 items through which problems associated with alcohol consumption are detected, by means of a Likert-type scale from 0 to 4.

A score of 0 means that there is no alcohol consumption, scores between 1 and 7 show a pattern of risk consumption, scores between 8 and 19 indicate harmful consumption, and scores of 20 to 40 are recognized as a pattern of harmful consumption and possible alcohol dependence [21].

**⚜** Problems associated with cannabis were assessed through the Spanish version of the Cannabis Abuse Screening Test (CAST) [18], which consists of 6 items through which the most common problems associated with cannabis use are detected, by means of a Likert-type scale from 0 to 4 (0 = never; 4 = very often).

Scores on the CAST questionnaire can range from 0 points to 24 points, so a low risk of problems with cannabis is at scores less than or equal to 3, moderate risk encompasses scores between 4 and 6, and high risk or severe addiction are scores greater than or equal to 7 [19].

**⚜** Sexual function was assessed by means of the Spanish version of the Changes in Sexual Functioning Questionnaire Short-Form (CSFQ-14) [17], which consists of 14 items, for both men and women, in which sexual function was assessed by means of desire, arousal and orgasm, through a Likert-type scale from 1 and to 5, where a higher score reflects better sexual function. For the interpretation of the questionnaire, it was considered that the total score of CSFQ-14 ranges from 14 to 70, where a higher score indicates better sexual function. The desire scale scores range from 5 to 25, and the arousal and orgasm scale scores range from 3 to 15. If the total CSFQ-14 questionnaire scores for men are less than or equal to 47 and less than or equal to 41 for women, they suggest global sexual dysfunction [17].

### 2.4. Ethical Considerations

Ethical approval was granted by the Ethics Committee of the Department of Nursing, Physiotherapy and Medicine at the University of Almeria (EFM 70/2020). The study was conducted in compliance with the Declaration of Helsinki and the ethical principles of biomedical research. The data collection was designed to ensure confidentiality and anonymity, and participants provided informed consent before conducting the study with the possibility to withdraw at any time.

### 2.5. Data Analysis

The IBM SPSS Statistics 23.0 program (Windows version, IBM, Armonk, NY, USA) was used to conduct the data analysis, and the level of significance was set at *p* ≤ 0.05. A descriptive statistical analysis was performed in which quantitative variables were expressed as means (M) and standard deviations (SD) or median, range (maximum and minimum values) based on their distribution and categorical variables, using a table of frequencies and percentages. A normality test was performed with the Kolmogorov–Smirnov test. To describe the study population, correlations between cannabis and alcohol consumption and the different variables were carried out. The Student’s *t*-test or one-way ANOVA in the parametric analysis or Mann–Whitney U or Kruskal–Wallis in the nonparametric analysis were used to compare the total score of the sexual function questionnaire and its subscales between cannabis and alcohol consumers and non-consumers.

## 3. Results

A total of 298 participants initially completed the questionnaire; however, 24 individuals were excluded because they did not meet the selection criteria (consumers of other substances, for example, heroin and opioids (*n* = 19); and presence of depression (*n* = 5)). Among the final 274 participants, 67.52% (*n* = 185) identified as female, while 32.48% (*n* = 89) identified as male. The participants were young adults aged 18 to 30, with a mean age of 21.89 years (SD = 2.23). Overall, 46% of the participants were not at risk for alcohol problems, while 41.6% were likely to have a moderate level of alcohol problems. Although slightly more than half of the sample (63.9%) reported no problems with cannabis use, the probability of developing a severe addiction was 23.7%. In terms of sexual function, only 4% of the participants indicated sexual dysfunction, while the remaining 96% reported none. The sociodemographic characteristics of the participants are summarised in Table 2.

Regarding sexual function, statistically significant differences (*p* < 0.05) were observed in both the total score of the questionnaire and the subscales of arousal and orgasm, with a higher mean score in cannabis users compared to nonusers. In the desire subscale, however, no differences were found (*p* > 0.05) (Table 3).

Besides this, the total score in the sexual function and arousal subscales were higher among severe cannabis consumers compared to non-consumers (*p* < 0.05), while no significant differences (*p* > 0.05) were found in the desire and orgasm subscales based on the amount of cannabis consumed by the participants (Table 4). No significant statistical differences were found between the sexes in sexual function scores or subscales (*p* > 0.05).

In terms of alcohol use, no significant differences (*p* > 0.05) in sexual function and the subscales measured were found between drinking and non-drinking participants. However, there were differences based on alcohol consumption (*p* < 0.05). Participants who reported heavy drinking scored higher on the total sexual function questionnaire and the arousal subscale than those who did not drink. Moreover, high-consumption participants had significantly higher total questionnaire and orgasm subscale scores than moderate-consumption participants (*p* < 0.05), while participants at high risk for alcohol use had significantly higher scores than those who reported alcohol dependence (*p* < 0.05). Having said that, no statistically significant differences in the desire subscale were found between participants who consumed alcohol and those who did not (*p* > 0.05) (Table 5). No significant statistical differences were found between men and women in sexual function or in the subscales (*p* > 0.05).

## 4. Discussion

The aim of this study was to analyse the effects of cannabis and alcohol use on sexual function in young people aged 18 to 30 years. It is well-known that the young population consumes both substances at a higher rate than others; therefore, it is of particular interest to understand the influence of these drugs on sexual activity, especially as the number of infections and sexually transmitted diseases among this population rises. In this context, the findings of this study revealed a higher score in sexual function, as well as arousal and orgasm, in subjects at risk of having cannabis-related problems and risk of addiction associated with alcohol consumption. Several studies have indicated the beneficial effects of using cannabis or alcohol to lessen emotions of anxiety and shame during sexual interactions, attaining physiological and psychoactive effects [22,23,24].

To date, there has been increasing interest in the influence of cannabis use on sexual function. Our findings indicate that young people who use cannabis frequently, regardless of gender, have better overall sexual function. These results are consistent with previous findings involving 216 people, both men and women, with an average age of 29.9 years, who used cannabis to improve their sexual experience. The effects of cannabis on heightened perceptions, time distortion, relaxation, and decreased inhibition were hypothesised as explanations for this improvement in sexual function [24]. In a similar line, another study [25] has found that women who reported more cannabis use showed higher sexual function scores, as well as desire, arousal, orgasm, and satisfaction. On the other hand, other researchers stated that the majority of women who used marijuana before sex reported positive sexual effects in the domains of overall sexual satisfaction, desire, orgasm and improvement in sexual pain but not in lubrication [13]. There are potential ties between cannabis and sexual health in men, where cannabis may have peripheral antagonistic effects on erectile function by stimulating specific receptors in cavernous tissue [26], thus linking cannabis use to worse erectile function or a higher level of follicle-stimulating hormone [15]. However, a recent study in males identified a connection between increased cannabis use and increased sexual function, as well as higher sexual satisfaction and a decreased prevalence of erectile dysfunction [27]. In this vein, a previous study found that cannabis users have higher sexual frequency than non-users as well as a higher score on the sexual health inventory and serum testosterone, among others [28].

Notwithstanding the benefits reported of cannabis consumption in our results and others, it is important to keep in mind that drug use is associated with risky sexual behaviours such as unprotected sex and the appearance of sexually transmitted infections [29,30], leading to careless and unsafe sexual encounters [31]. These high-risk attitudes are frequently associated with increased relaxation, euphoria, disinhibition, decreased self-control, and decreased risk perception caused by psychoactive substances, which cause users to be less cautious and to forget the importance of safe sex [32]. It should also be noted that for the large number of younger populations, alcohol and tobacco consumption is closely related to night-time recreational environments, which are a key element for socialization and where the consumption of different substances can lead to altered decision-making regarding sexual relations, associated with an increase in the likelihood of engaging in risky sexual behaviours [31,32,33].

Similarly, certain contradictions have been observed in some studies regarding the influence of alcohol consumption on sexuality. These studies, like ours, show that alcohol consumption improves sexual function, arousal, and orgasm in participants at no or intermediate risk for alcohol problems, as well as those at high risk. Under the influence of the primary substance of abuse, most patients with a substance use disorder, including alcohol, reported improvements in sexual pleasure, arousal, and sexual behaviour [31]. Despite this, another study found that alcohol consumption was associated with significantly worse sexual functioning in its male participants, with erectile dysfunction being prevalent in the majority of frequent alcohol consumers, and that alcohol consumption was associated with a higher risk of sexual dysfunction in those at high risk [34]. One possible explanation for this would be the fact that the participants in their study were older; the current study focused on a population between the ages of 18 and 30, where erectile dysfunction is less common. As for female participants, the current study found no significant differences in sexual function among women at risk for alcohol-related problems, despite research indicating a positive relationship between alcohol consumption and sexual function in a large sample of women with a mean age of 51.8 years [35].

### Limitations

Nevertheless, some limitations need to be considered. The number of participants in this study was relatively small; therefore, the study findings should be interpreted cautiously. These findings are based on a convenience sample, and some participants may have refused to participate because cannabis use is currently illegal in Spain, and their social acceptability may have resulted in a denial of cannabis use. Rather than concluding the issue, many questions arise from this study that need to be answered. Future research could delve into cannabis and alcohol consumers’ perceptions of their sexuality to gain a better understanding of the issue, as well as the types of relationships (long-term, sporadic, unstable, etc.) that frequent drug users have and whether there is an association between their use and the type of relationship they have. Moreover, the relationship between anxiety, cannabis consumption, and sexual function deserves further investigation. Lastly, it would be particularly interesting to investigate the long-term influence on sexuality of males and females who consume or have consumed alcohol and cannabis on a regular basis, since some of the problems observed do not have short-term consequences but rather medium- or long-term consequences.

## 5. Conclusions

Sexual function is improved in young people who are high-risk cannabis consumers with a moderate risk of alcohol use, resulting in increased desire, arousal, and orgasm. This improvement is usually associated with a reduction in anxiety and shame, which facilitates sexual relationships. However, according to the literature, this increase in sexual function is generally accompanied by unsafe sexual behaviours, given that the use of drugs, notably alcohol and cannabis, in recreational nightlife settings is highly normalized and ingested in large quantities. Thus, further information and training on the sexual risks involved with the use of substances such as cannabis and alcohol is required, particularly for young people, who are the population most vulnerable to sexual risk behaviours and health-related problems associated with drug use. Additionally, sexual education for the younger population must incorporate strategies and education to lessen anxiety and shame during sex encounters.

## Figures and Tables

**Table 1:** Variables and data collection tools.

Variables	Collection Tools
Sociodemographic variables	Ad-hoc questionnaire
Sexual function	The Spanish version of the CSFQ-14 (Changes in Sexual Functioning Questionnaire Short-Form) [17]
Cannabis-related problems	The Spanish version of the CAST (Cannabis Abuse Screening Test) [18,19]
Alcohol-related problems	The Spanish version of the AUDIT (Alcohol Use Disorders Identification Test) [20,21]

**Table 2:** Sociodemographic characteristics of the participants.

Variables	M (SD)/*n* (%)
**Age, *x̄* (SD)**	21.89 (2.23)
**Sex, *n* (%)**	
Female	185 (67.5%)
Male	89 (32.5%)
**Level of studies, *n* (%)**	
Primary Education or equivalent	1 (0.4%)
Compulsory Secondary Education or equivalent	7 (2.6%)
Post-Compulsory Education or equivalent	24 (8.8%)
Higher Education	212 (77.4%)
Vocational Training	30 (10.9%)
**Sexual identity, *n* (%)**	
Heterosexual	246 (89.8%)
Homosexual	14 (5.1%)
Bisexual	14 (5.1%)
**Alcohol use (AUDIT), *n* (%)**	
Lower risk	126 (46.0%)
Increasing risk	114 (41.6%)
Higher risk	17 (6.2%)
Possible dependence	17 (6.2%)
**Cannabis use (CAST), *n* (%)**	
Lower risk	175 (63.9%)
Moderate risk	34 (12.4%)
Higher risk	65 (23.7%)
**Sexual function (CSFQ-14), *n* (%)**	
Sexual dysfunction	11 (4%)
No sexual dysfunction	263 (96.0%)
**Sexual function (CSFQ-14), *n* (SD)**	53.82 (6.45)
Desire	18.07 (2.94)
Arousal	12.21 (2, 08)
Orgasm	11.38 (2.30)

**Table 3:** The relationship between cannabis use and sexual function.

Variables	Cannabis Use			
	No Risk	Risk	F	*p*
Sexual function	52.93 ± 6.76	55.25 ± 5.64	2.68	0.01
Desire	17.70 ± 3.21	18.37 ± 3.37	0.18	0.10
Arousal	12.00 ± 2.16	12.57 ± 1.87	1.20	0.03
Orgasm	11.26 ± 2.37	11.78 ± 2.16	1.65	0.03

The table shows the scores, with “±” values indicating the SD.

**Table 4:** The relationship between cannabis use and sexual function according to categories.

Variables	Cannabis Use				
	No Risk	Moderate Risk	High Risk	F	*p*
Sexual function	52.93 ± 6.76 *	54.24 ± 5.44	55.78 ± 5.69 *	4.85	0.008
Desire	17.70 ± 3.21	17.97 ± 4.33	18.58 ± 2.75	1.73	0.18
Arousal	12.00 ± 2.16 *	12.12 ± 1.82	12.80 ± 1.86 *	3.63	0.03
Orgasm	11.13 ± 2.37	11.65 ± 2.33	11.85 ± 2.08	2.63	0.07

The table shows the scores, with “±” values indicating the SD. * indicates significant differences between cannabis use and sexual function.

**Table 5:** The relationship between alcohol use and sexual function.

Variables	Alcohol Use					
	Lower Risk	Increasing Risk	Higher Risk	Possible Dependence	F/H	*p*
Sexual function	53.90± 6.35 *	52.74 ± 6.71 †‡	57.24 ± 4.60 *†	56.24 ± 5.56 ‡	3.54	0.015
Desire	17.94 ± 3.67	17.62 ± 2.96	19.06 ± 2.95	19.00 ± 2.06	1.62	0.19
Arousal	12.12 ± 1.92 *	12.03 ± 2.29	13.41 ± 0.94 *	12.82 ± 2.10	9.69 §	0.02
Orgasm	11.44 ± 2.29	11.01 ± 2.39 *	12.47 ± 1.66 *	12.06 ± 2.11	2.80	0.04

The table shows the scores, with “±” values indicating the SD. *, † and ‡ indicate significant differences between alcohol use and sexual function. ^§^ statistical differences between arousal subscale and alcohol use were assessed using Kruskal–Wallis test (*p* < 0.05).

## Data Availability

The data that support the findings of this study are available on request from the corresponding author. The data are not publicly available due to privacy or ethical restrictions.

## References

[1] Gentzke A.S., Wang T.W., Jamal A., Park-Lee E., Ren C., Cullen K.A., Neff L. (2020). Tobacco product use among middle and high school students—United States, 2020. Morb. Mortal. Wkly. Rep..

[2] La Fauci V., Squeri R., Spataro P., Genovese C., Laudani N., Alessi V. (2019). Young people, young adults and binge drinking. J. Prev. Med. Hyg..

[3] Páramo M.F., Cadaveira F., Tinajero C., Rodríguez M.S. (2020). Binge drinking, cannabis co-consumption and academic achievement in first year university students in Spain: Academic adjustment as a mediator. Int. J. Environ. Res. Public Health.

[4] Baumeister S.E., Baurecht H., Nolde M., Alayash Z., Gläser S., Johansson M., Amos C.I., Johnson E.C., Hung R.J. (2021). Cannabis use, pulmonary function, and lung cancer susceptibility: A mendelian randomization study. J. Thorac. Oncol..

[5] Husain K., Ansari R.A., Ferder L. (2014). Alcohol-induced hypertension: Mechanism and prevention. World J. Cardiol..

[6] Rojewski A.M., Tanner N.T., Dai L., Ravenel J.G., Gebregziabher M., Silvestri G.A., Toll B.A. (2018). Tobacco dependence predicts higher lung cancer and mortality rates and lower rates of smoking cessation in the National Lung Screening Trial. Chest.

[7] Lawn W., Aldridge A., Xia R., Winstock A.R. (2019). Substance-linked sex in heterosexual, homosexual, and bisexual men and women: An online, cross-sectional “Global Drug Survey” report. J. Sex. Med..

[8] Walker G.M., Walker R.S.K. (2018). Enhancing yeast alcoholic fermentations. Adv. Appl. Microbiol..

[9] Gilmore A.K., George W.H., Nguyen H.V., Heiman J.R., Davis K.C., Norris J. (2013). Influences of situational factors and alcohol expectancies on sexual desire and arousal among heavy-episodic drinking women: Acute alcohol intoxication and condom availability. Arch. Sex. Behav..

[10] George W.H., Davis K.C., Heiman J.R., Norris J., Stoner S.A., Schacht R.L., Hendershot C.S., Kajumulo K.F. (2011). Women’s sexual arousal: Effects of high alcohol dosages and self-control instructions. Horm. Behav..

[11] Cartledge J., Minhas S., Eardley I. (2001). The role of nitric oxide in penile erection. Expert Opin. Pharmacother..

[12] Wang X.M., Bai Y.J., Yang Y.B., Li J.H., Tang Y., Han P. (2018). Alcohol intake and risk of erectile dysfunction: A dose–response meta-analysis of observational studies. Int. J. Impot. Res..

[13] Lynn B.K., López J.D., Miller C., Thompson J., Campian E.C. (2019). The relationship between marijuana use prior to sex and sexual function in women. Sex. Med..

[14] Smith A.M.A., Ferris J.A., Simpson J.M., Shelley J., Pitts M.K., Richters J. (2010). Cannabis use and sexual health. J. Sex. Med..

[15] Khan N., Shah M., Malik M.O., Badshah H., Habib S.H., Shah I., Shah F.A. (2021). The effects of tobacco and cannabis use on semen and endocrine parameters in infertile males. Hum. Fertil..

[16] von Elm E., Altman D.G., Egger M., Pocock S.J., Gøtzsche P.C., Vandenbroucke J.P. (2008). The Strengthening the Reporting of Observational studies in Epidemiology (STROBE) statement: Guidelines for reporting observational studies. J. Clin. Epidemiol..

[17] Garcia-Portilla M.P., Saiz P.A., Fonseca E., Al-Halabi S., Bobes-Bascaran M.T., Arrojo M., Benabarre A., Goikolea J.M., Sanchez E., Sarramea F. (2011). Psychometric properties of the spanish version of the changes in sexual functioning questionnaire short-form (CSFQ-14) in patients with severe mental disorders. J. Sex. Med..

[18] Fernández-Artamendi S., Fernández-Hermida J.R., García-Cueto E., Secades-Villa R., García-Fernández G., Barrial-Barbén S. (2012). Spanish adaptation and validation of The Adolescent-Cannabis Problems Questionnaire (CPQ-A). Adicciones.

[19] Casajuana Kögel C., López-Pelayo H., Oliveras C., Colom J., Gual A., Balcells-Oliveró M.M. (2021). The relationship between motivations for cannabis consumption and problematic use. Adicciones.

[20] García-Carretero M.A., Novalbos-Ruiz J.P., Martínez-Delgado J.M., O’Ferrall-González C. (2016). Validation of the alcohol use disorders identification test in university students: AUDIT and AUDIT-C. Adicciones.

[21] Tejedor-Cabrera C., Cauli O. (2019). Alcohol and cannabis intake in nursing students. Medicina.

[22] Parent N., Coulaud P.J., Amirie M., Ferlatte O., Knight R. (2021). Cannabis use and mental health among young sexual and gender minority men: A qualitative study. Int. J. Drug Policy.

[23] Parent N., Ferlatte O., Milloy M.J., Fast D., Knight R. (2021). The sexualised use of cannabis among young sexual minority men: “I’m actually enjoying this for the first time”. Cult. Health Sex..

[24] Wiebe E., Just A. (2019). How cannabis alters sexual experience: A survey of men and women. J. Sex. Med..

[25] Kasman A.M., Bhambhvani H.P., Wilson-King G., Eisenberg M.L. (2020). Assessment of the association of cannabis on female sexual function with the female sexual function index. Sex. Med..

[26] Pizzol D., Demurtas J., Stubbs B., Soysal P., Mason C., Isik A.T., Solmi M., Smith L., Veronese N. (2019). Relationship between cannabis use and erectile dysfunction: A Systematic review and meta-analysis. Am. J. Mens. Health.

[27] Bhambhvani H.P., Kasman A.M., Wilson-King G., Eisenberg M.L. (2020). A survey exploring the relationship between cannabis use characteristics and sexual function in men. Sex. Med..

[28] Shiff B., Blankstein U., Hussaen J., Jarvi K., Grober E., Lo K., Lajkosz K., Krakowsky Y. (2021). The impact of cannabis use on male sexual function: A 10-year, single-center experience. Can. Urol. Assoc. J..

[29] Slavin M.N., Hochstatter K., Kraus S.W., Earleywine M., El-Bassel N. (2021). Associations between cannabis use and sexual risk behavior among women under community supervision: A brief report. Int. J. Sex. Health.

[30] Clark D.A., Arterberry B.J., Walton M.A., Cunningham R.M., Goldstick J.E., Zimmerman M.A., Davis A.K., Bonar E.E. (2021). Examining same-day associations between cannabis use motives and condom use in urban emerging adults: A brief report. J. Stud. Alcohol Drugs.

[31] Bosma-Bleeker M.H., Blaauw E. (2018). Substance use disorders and sexual behavior; the effects of alcohol and drugs on patients’ sexual thoughts, feelings and behavior. Addict. Behav..

[32] Lomba L., Apostolo J., Mendes F. (2009). Drugs and alcohol consumption and sexual behaviours in night recreational settings in Portugal. Adicciones.

[33] Shamloul R., Bella A.J. (2011). Impact of cannabis use on male sexual health. J. Sex. Med..

[34] Navarro A.D., Marín G.H. (2012). Alcohol, función sexual y masculinidad. Rev. Cuba. Med. Gen. Integr..

[35] Kling J.M., Sidhu K., Rullo J., Mara K.C., Frohmader Hilsaca K.S., Kapoor E., Faubion S.S. (2019). Association between alcohol use and female sexual dysfunction from the data registry on experiences of aging, menopause, and sexuality (DREAMS). Sex. Med..

